# Discriminant Analysis of Defective and Non-Defective Field Pea (*Pisum sativum L*.) into Broad Market Grades Based on Digital Image Features

**DOI:** 10.1371/journal.pone.0155523

**Published:** 2016-05-13

**Authors:** Linda S. McDonald, Joseph F. Panozzo, Phillip A. Salisbury, Rebecca Ford

**Affiliations:** 1 Agriculture Research, Department of Economic Development, Jobs, Transport and Resources, Horsham, Victoria, Australia; 2 Faculty of Veterinary and Agricultural Sciences, The University of Melbourne, Parkville, Victoria, Australia; 3 School of Natural Sciences, Griffith University, Nathan, Queensland, Australia; Agricultural University of Athens, GREECE

## Abstract

Field peas (*Pisum sativum L*.) are generally traded based on seed appearance, which subjectively defines broad market-grades. In this study, we developed an objective Linear Discriminant Analysis (LDA) model to classify market grades of field peas based on seed colour, shape and size traits extracted from digital images. Seeds were imaged in a high-throughput system consisting of a camera and laser positioned over a conveyor belt. Six colour intensity digital images were captured (under 405, 470, 530, 590, 660 and 850nm light) for each seed, and surface height was measured at each pixel by laser. Colour, shape and size traits were compiled across all seed in each sample to determine the median trait values. Defective and non-defective seed samples were used to calibrate and validate the model. Colour components were sufficient to correctly classify all non-defective seed samples into correct market grades. Defective samples required a combination of colour, shape and size traits to achieve 87% and 77% accuracy in market grade classification of calibration and validation sample-sets respectively. Following these results, we used the same colour, shape and size traits to develop an LDA model which correctly classified over 97% of all validation samples as defective or non-defective.

## Introduction

Field pea (*Pisum sativum L*.) is generally traded based on broad quality grades, each of which has its own market niche. Grades are determined subjectively and often classified inconsistently between the grain sellers and buyers, leading to trading disputes. Khan and Croser [1] identified five broad types of field pea (yellow, marrowfat, dun, green/blue and maple) and six quality traits which heavily influence their marketing; admixture levels, insect damage, seed colour, seed size, seed cleanliness and product uniformity. Historically, these traits are based on appearance and are assessed visually. As such, the trading value of field pea (like most pulse grains) is subjectively determined. There is an opportunity, therefore, for objective measurement of grain products using colour grading systems or machine vision to reduce the potential for inconsistent assessment.

Within the grains research field, several studies have been conducted on the application of machine vision systems to quantitatively determine characteristics related to grain quality. Zapotoczny and Majewska [2] investigated the measurement of wheat colour, of both the endosperm and grain coat, by machine vision. Fundamental size traits, such as grain length, width and volume, have been modelled in various studies [3–6], as well as shape of grains [7–9]. Further to grain size, shape and colour analysis, machine vision studies have also been applied to assess traits which impact on grain processing, such as chalkiness in rice [10, 11], performance of wheat samples through a dockage tester (Paliwal, Visen et al. 2003) and distribution of grain size [12, 13], which impacts on milling efficiency.

Machine vision sytems have also been used in the grains industry for colour-based grading and identifying defects and seed damage. While 2-dimensional colour, size and shape traits are the most commonly used, more recent focus has been on expanding the range of image traits to include textural, morphological, and wavelet features, enabling a suite of measurments from each image and contributing to an increased efficency and justification of the capital expenditure in setting up digital image technology. Anami and Savakar [14] provided a summary on some of the most common feature extraction methods used in the analysis of grains, fruits and flowers. Choudhary, Paliwal [15] developed a model to classify cereal grains into grain type (wheat, rye, barley and oats) and reported that the combination of morphological, colour, textural as well as wavelet features gave the best results for classification. A number of studies have identified type and extent of cereal grain [16, 17] and legume grain defects [18–22] through digital image analysis (DIA). Key to all of these assessments were the extracted image features chosen to inform statistical and analytical models for measuring and classifying the grain quality traits. Zheng, Sun [23] provided an overview of textural features for assessing food quality by DIA and identified the two most commonly adopted classification methods as Statistical Learning (SL), for example discriminant analysis and Bayesian learning, and Artificial Neural Networks (ANN). Choice of image features and classification method is important for ensuring accuracy and efficiency of field pea broad market grade assessment by DIA.

Field-pea market grades are classified by key visual-characteristics of seed shape, size and colour of seed coat and cotyledon. However, all of these characteristics can be altered by various seed defects. For example, diseases and/or weather damage can cause discoloration, deformation and/or shrivelling of seed. Therefore the development of a robust model to classify broad market grades, of defective as well as non-defective grain, should use image features which best represent these key visual characteristics. While two-dimensional images of grain can contribute a large number of helpful size, shape and colour classification features, grain surface height information is also useful for measuring traits such as dimpling and correcting variations in colour intensity readings due to variable surface height. In this study, we developed models to classify field pea into broad market grades and investigated the impact of grain defects on model performance. We extracted a number of colour, shape and size (including laser-measured seed height) features from images of field pea seed. These features were used to build Linear Discriminant Analysis (LDA) models to classify the seed into common Australian market grades; White Pea, Blue Pea, Mottled-Dun Pea, Kaspa-Dun Pea, Green-Dun Pea, Yellow-Forage Pea, Marrowfat Pea, and Kaspa-Type Pea.

## Materials & Methods

### Sample collection and classification

Field pea seed samples were sourced from the 2013/14 and 2014/15 harvests of the breeding and agronomy trials undertaken by the Department of Economic Development, Jobs, Transport and Resources based in Horsham, Victoria, Australia. Samples were considered as defective when at least 75% of the seed were either disease-stained, weather damaged, insect damaged or broken. Samples were considered as non-defective if less than 5% of the seeds were impacted by defects. The distinction between defective and non-defective samples was intended to improve clarity in observing the impact of defects on classification models. There were 239 non-defective grain samples and 78 defective samples selected at random within these two categories. Each field pea sample was manually classified into one of eight market grades (S1 Fig and S1 Table). Samples were then divided into calibration and validation sets (Table 1); a Calibration set of 175 samples (including 39 defective samples) and a Validation set of 142 samples (including 39 defective samples).

**Table 1:** Field pea calibration and validation sets.

Market Grade	All Calibration Samples	Defective Samples in Calibration Set	All Validation Samples	Defective Samples in Validation set
White	50	4	45	4
Blue	31	0	44	0
Mottled Dun	7	4	5	4
Kaspa Dun	13	7	8	6
Green Dun	16	0	5	1
Yellow Forage	5	5	6	6
Marrowfat	6	0	2	0
Kaspa type	47	19	27	18
	**175**	**39**	**142**	**39**

### Model development

**Fig 1:**
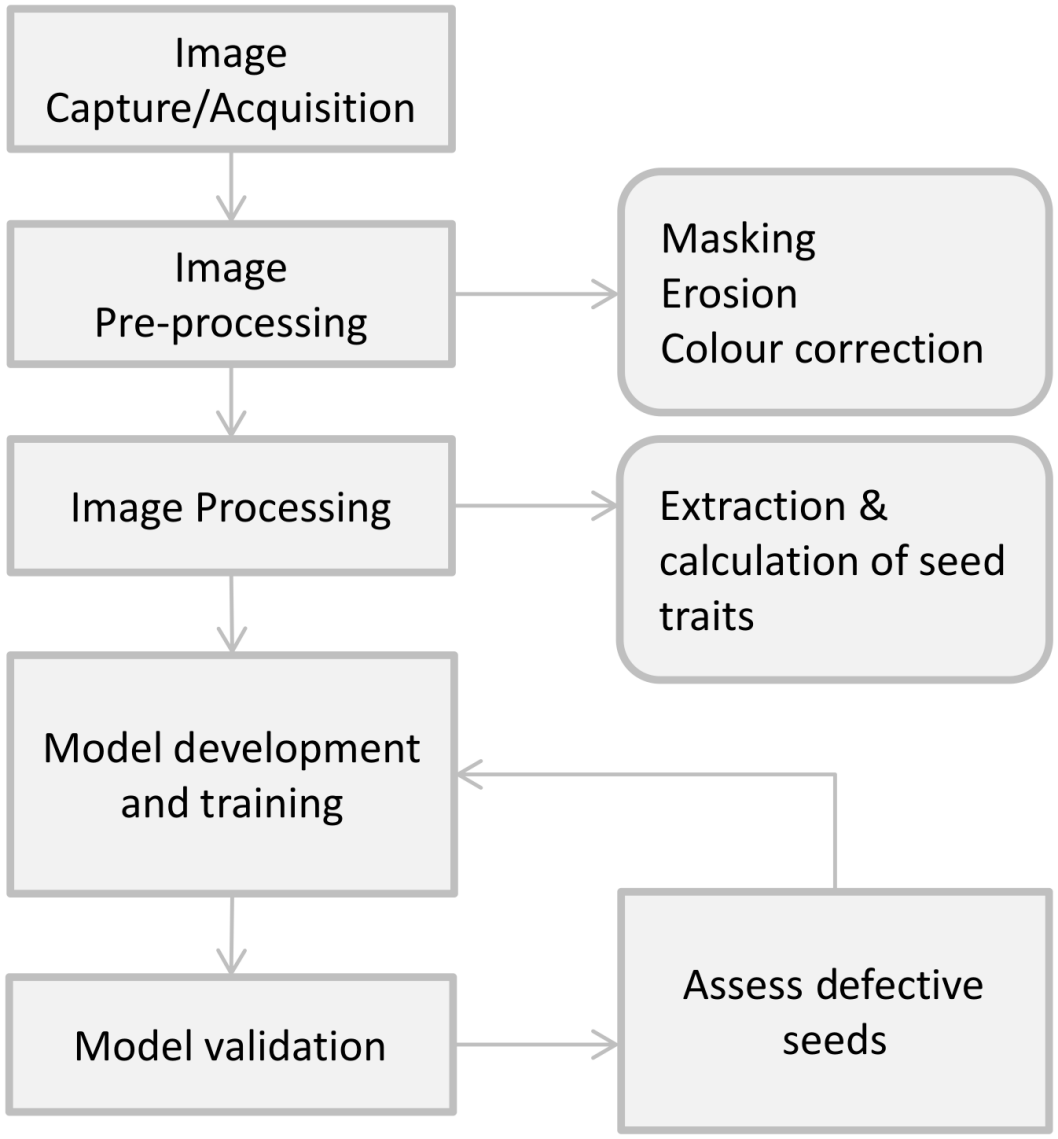
Model development flow chart.

Model development is depicted in Fig 1 and detailed below.

### Image Capture

Images were captured through an EyeFoss^™^ (FOSS Analytical, Hoganas, Sweden) as described by LeMasurier, Panozzo [13]. For each individual field pea seed, the EyeFoss^™^ captured six colour intensity images (under LED light sources of 405, 470, 530, 590, 660 and 850nm) and simultaneously measured surface height, by laser, at each pixel location. Colour intensity and height images were stored as double precision, floating point number arrays.

### Image Pre-processing

Each image was segmented, using the method described by LeMasurier, Panozzo [13], to create a binary image mask (**M**_**1**_), which was used to detect the seed boundary and to measure size and shape characteristics. A second binary mask image (**M**_**2**_) was created by setting a threshold of 20 units on the heights image. **M**_**2**_ was used in the calculation of colour and height traits to avoid interference from seed boundary values where height was near zero and colour intensity values were affected by shadowing.

### Image Processing

**Fig 2:**
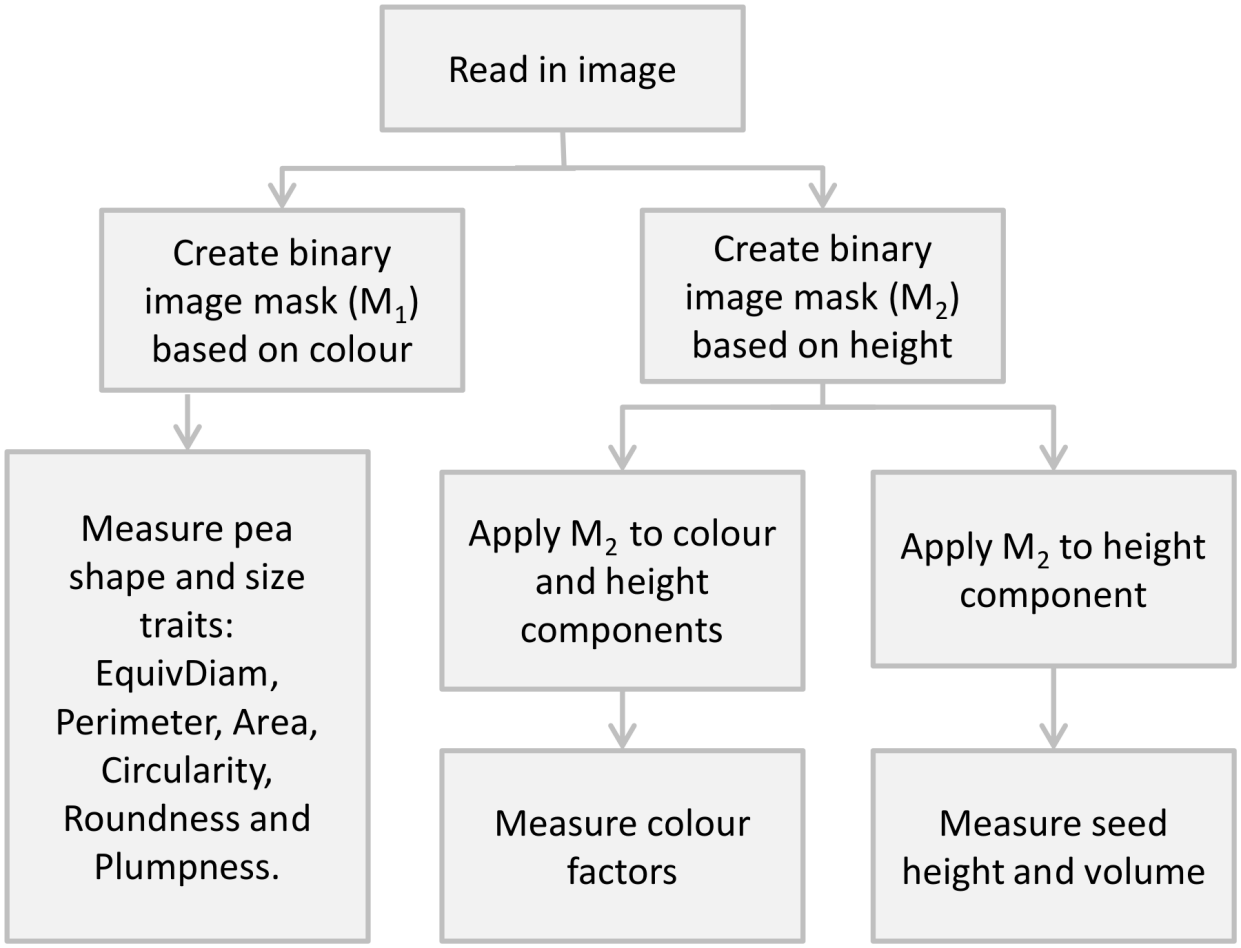
Image pre-processing and processing flow chart.

Single-seed images were processed according to the flow chart in Fig 2. All single-seed features extracted from the images are outlined in Table 2. The median value, of each feature across all seeds in each sample, was taken as the feature value for that sample. Feature values were standardised to have zero mean and unit standard deviation across the Calibration Set of samples.

**Table 2:** Seed characteristics extracted through image processing.

Single Seed Features	Measurement/Calculation
Violet colour factor	Apply the mask **M**_**2**_ to both the height image and the violet (405nm) colour intensity image. Within the seed region, divide each pixel colour value by the corresponding height value^a^. The violet factor was taken as the median of all corrected pixel colour values.
Blue colour factor, Green colour factor, Orange colour factor, Red colour factor and NIR colour factor	As for Violet colour factor but calculate on the blue (470nm), green (530nm), orange (590nm), red(660nm) and NIR (850nm) intensity images, respectively.
Seed height	Apply the mask **M**_**2**_ to the height image and take the median value within the seed region.
Equivalent diameter, Area and Plumpness	As detailed by LeMasurier, Panozzo [13]
Perimeter	Number of pixels in seed boundary
Volume	Sum of all values within seed region of heights image (after applying **M**_**1**_)
Circularity	(area x 4) / (Equivalent diameter x Perimeter)

^a^ Pixel colour values were divided by height values to remove variations in colour intensities due to surface height of the seed. Initial observations of uniformly coloured seeds indicated that colour intensity varied linearly with grain surface height as measured by laser

### Model development

Market grades of field pea are defined by visual traits (S1 Table), therefore all image features (Table 2) were deliberately related directly to colour, size or shape of the seeds. Features were tested to ensure linear independence and then selected for model training based on prior knowledge of dominant discriminating traits of market grades. Therefore preference was given first to colour, then size and then shape features. Features were added one at a time to the model and those which did not improve accuracy of classifications were discarded. Models were trained such that they would require the minimum number of features to achieve greatest possible accuracy and robustness in predictions. Since there was clear visual distinction between field pea market grades (S1 Fig), it was assumed that, with the appropriate selection of image features, market grades would be linearly separable. LDA was therefore chosen as the classification method because of its relative simplicity and lower computational cost compared with other classification methods. The LDA models were constructed and analysed through Matlab (R2014b) with the Statistics Toolbox.

### Model 1 and 2

The first LDA model (Model 1) was trained on the non-defective samples of the Calibration Set and validated on the non-defective samples of the Validation Set. This was done to observe the greatest potential accuracy of predicting field pea market grades, since the market grades are defined by appearance of non-defective grain. The performance of this model was then tested on the defective samples of the Validation Set to observe the impact of seed defects on market grade assessment. A second LDA model (Model 2) was subsequently calibrated and validated, on the full Calibration Set and Validation Set respectively (including defective samples) to observe changes to classification robustness from Model 1. Features were chosen for Model 2 to give the simplest model with the most accurate predictions for both defective and non-defective field pea samples.

### Predicting defects

A difference in classification accuracy between Model 1 and Model 2, in assessment of the defective samples, was assumed to indicate that a third LDA model could be constructed based on the same input variables to distinguish defective from non-defective field pea samples.

## Results and Discussion

The three LDA models which were developed are outlined in Table 3 with their respective calibration and validation sample sets, input variables and output classes. The performance of each model is outlined in Table 4.

**Table 3:** Linear discriminant analysis models and parameters.

	Model 1	Model 2	Defect prediction Model
**Calibration samples**	Calibration Set excluding defective samples	Full Calibration Set	Full Calibration Set
**Validation samples**	Full Validation Set; Separately assessing non-defective then defective samples	Full Validation Set	Full Validation Set
**Input variables**	Blue, green, orange and red factors	As for Model 1 plus violet colour factor, equivalent diameter, circularity and plumpness	As for Model 2
**Classification Groups**	White, Blue, Mottled-Dun, Kaspa-Dun, Green-Dun, Marrowfat and Kaspa type	White, Blue, Mottled-Dun, Kaspa-Dun, Green-Dun, Yellow-Forage, Marrowfat and Kaspa type	Defective and non-defective

**Table 4:** Classification Rates of Models.

Prediction Model	% Accuracy in prediction of non-defective calibration samples (n = 136)	% Accuracy in prediction of defective calibration samples (n = 39)	% Accuracy in prediction of non-defective validation samples (n = 103)	% Accuracy in prediction of defective validation samples (n = 39)
Model 1	100	NA	100	69^a^
Model 2	100	87	100	77
Defect prediction model	100	100	97	100

^a^ This value does not include prediction of yellow forage peas as these were excluded from Model 1.

### Feature Selection

A one-way Multivariate Analysis of Variance (MANOVA) was performed on all extracted image features (Table 2) for the full set of calibration samples. Since the choice of features was based on the definitions of market grades, it was not surprising that the F statistic of each feature was significant (p <0.001), indicating that any of the features could in some way be useful for separating samples according to their market grade. All feature vectors were found to be linearly independent by Singular Value Decomposition (SVD). Prior knowledge of dominant visual traits in the definitions of field pea grades determined the order of feature selection. Preference was given first to colour, then size and then shape features.

### Model 1

**Fig 3:**
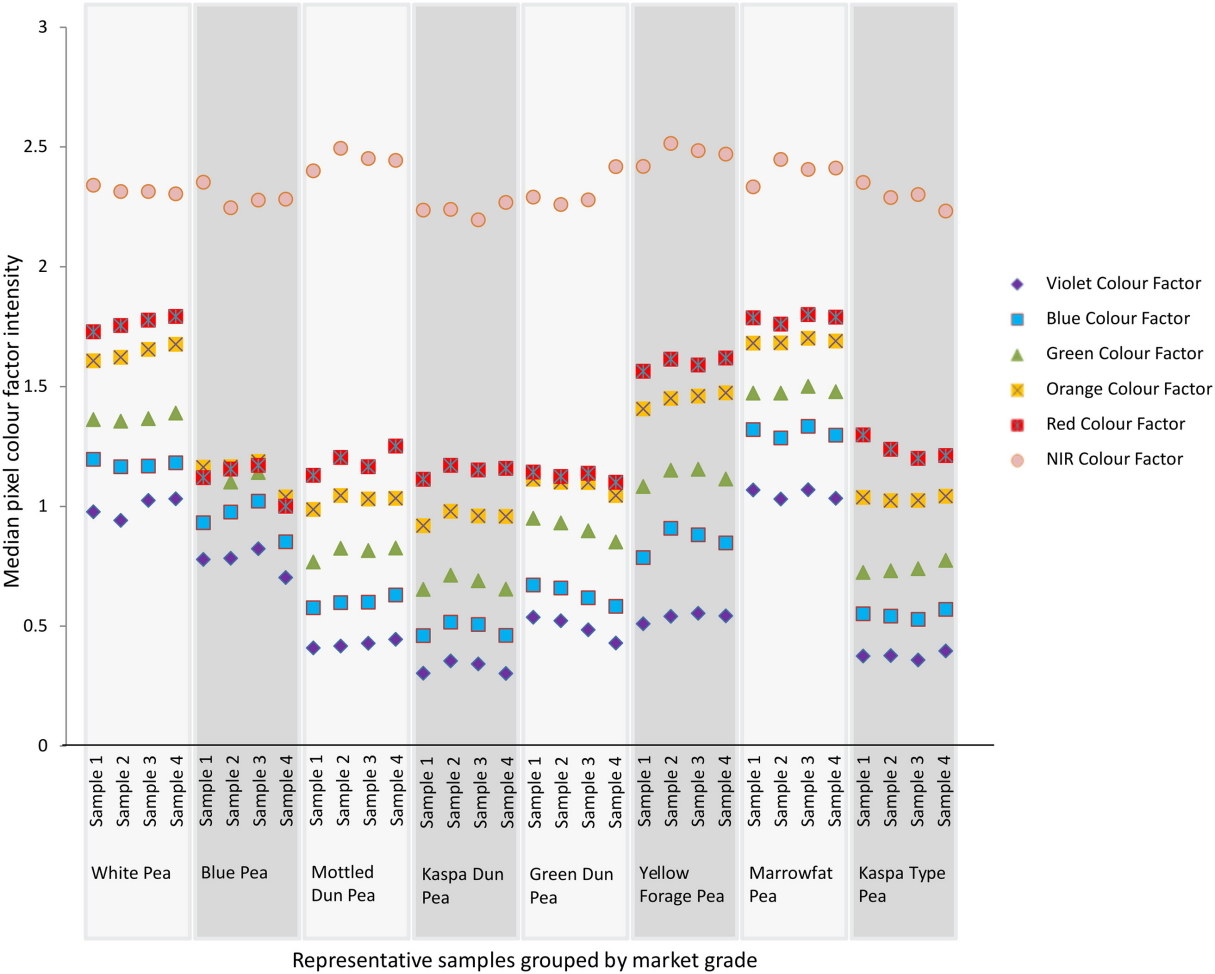
Colour factor variations between field pea market grades. Four representative samples from each market grade illustrate the variation in relative colour intensity factors. The violet, blue, green, orange, red and NIR colour factors for each sample are represented respectively by the violet diamonds, blue squares, green triangles, yellow squares, red squares and pink circles. These are the basis for predicting market grades through Model 1.

Image colour traits alone were sufficient inputs to classify all of the non-defective field pea samples (both calibration and validation sets) into correct market grades. Four of the six colour traits (red, orange, green and blue factors) gave the optimum combination of input variables. The NIR factor did not vary markedly between different field pea groups (Fig 3) and therefore did not impact on market grade predictions. Violet colour intensity varied substantially between different market grades but had little impact on the prediction of non-defective grain samples as there was sufficient information in the four mid-range wavelength colour factors. Model 1 excluded predictions of Yellow-Forage peas since all samples of forage peas were defective.

While non-defective peas were accurately classified into their appropriate market grade through Model 1, this was not the case for defective pea samples (Table 4). The majority of misclassified defective samples were from the Kaspa, Kaspa-Dun and Green-Dun groups, which were categorised as Mottled-Dun peas. This was not surprising, as disease staining, which can have similar dark toned patterns to seed coat speckling, was the most prevalent defect.

Defects other than disease staining also have impact on colour of the seeds. For example harvest damage or insect damage can expose areas of the cotyledon. One white pea sample was misclassified as a marrowfat type because the seed surface colour was affected by areas of seed coat detaching from the cotyledon. Though white pea can be similar in colour to marrowfat pea, they are very different in size and shape (Table 1).

The results of Model 1 indicated that colour features were significantly impacted by defects in the field peas since the accuracy in prediction of defective validation samples was much lower than for non-defective validation samples. Therefore Model 2 was constructed to include inputs of seed size and shape traits (equivalent diameter, circularity and plumpness) additional to the violet colour factor and the inputs of Model 1 (Table 3).

### Model 2

**Fig 4:**
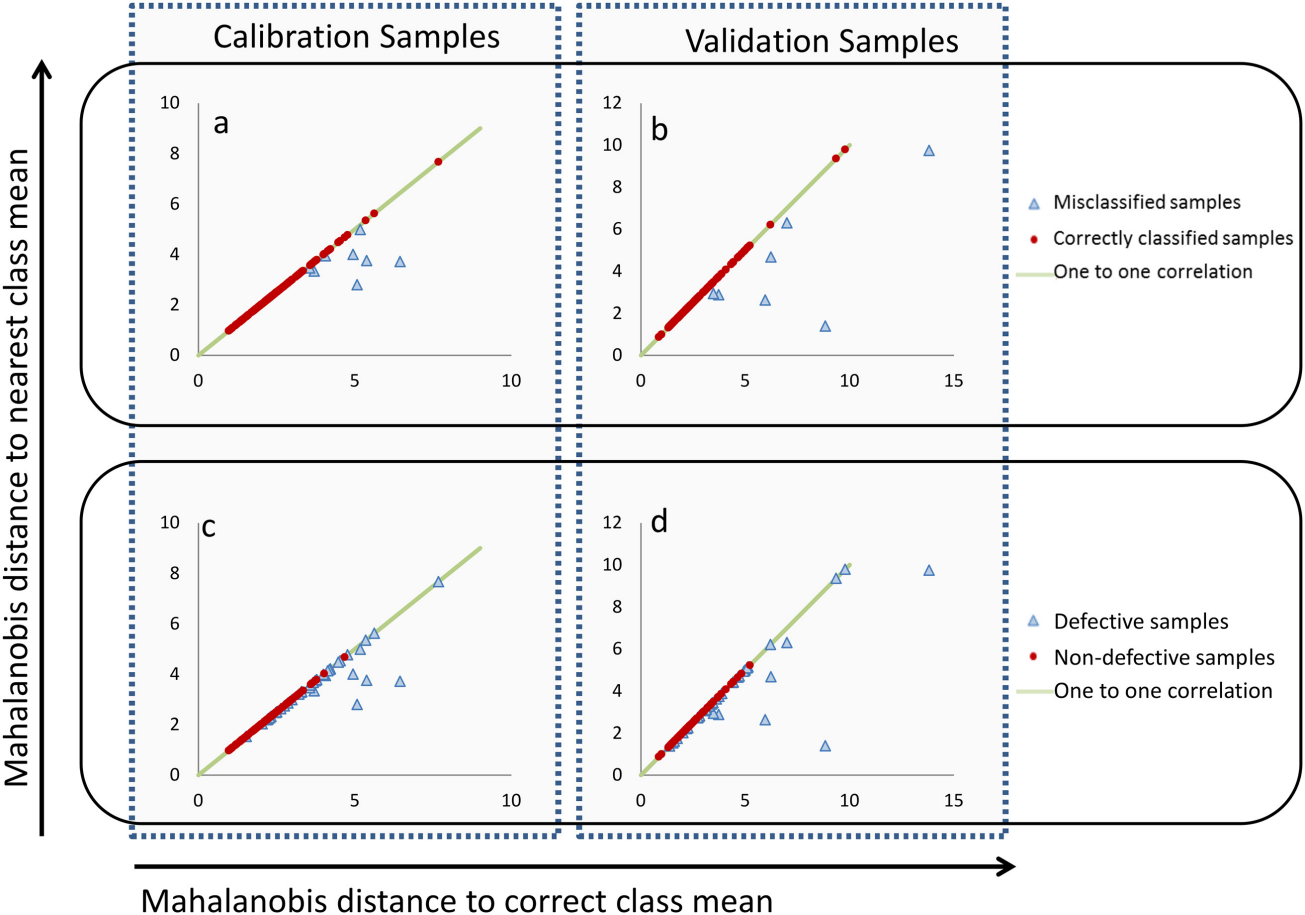
Performance of Model 2. (a) and (b) All samples that were correctly classified (red dots) fell along the one to one correlation line (green), i.e. the closest market grade mean was the correct market grade mean for that sample. All samples which did not lie on the green line (one to one correlation) were incorrectly classified (blue triangles). Plots (c) and (d) gave the same scatter plots as (a) and (b) but highlighted which samples were non-defective (red dots) and which were defective (blue triangles).

Model 2 maintained the accuracy of Model 1 in classifying market grades of non-defective samples and improved classification of defective sample sets (Table 4). Non-defective samples tended to lie closer, in terms of Mahalanobis distance, to their correct Market Grade mean than the defective samples (Fig 4c and 4d). In the Validation set, the mean Mahalanobis distance of defective and non-defective samples from their nearest class mean was 3.91±0.33 and 2.42±0.09 respectively. Therefore, the non-defective samples were more accurately predicted, and with greater confidence, than the defective samples.

Three defective samples that were correctly classified through Model 1, were then misclassified through Model 2. All three samples had both disease staining and insect damage. Two of the samples were white peas, misclassified as Yellow Forage peas, with seed smaller than the average size of White pea market grade. The third sample was a Mottled-Dun which was misclassified as a Kaspa-Dun type. In all three cases the exposed cotyledon appears to have affected model predictions by causing the average seed surface colour to be more yellow. Therefore, a method for identifying and removing areas of exposed cotyledon in seed images would be useful to improve market grade predictions based on seed coat appearance.

Of the defective samples that were initially misclassified through Model 1, more than 70% were subsequently classified correctly through Model 2. Most of these samples were defective due to disease staining, which did not appear to affect seed shape and size. This implied that the violet colour factor played a significant role in distinguishing disease staining apart from natural speckling. Nine of the defective samples, which had been misclassified through Model 1, remained misclassified through Model 2. These samples were predominantly disease stained Kaspa-Dun and Kaspa-Type samples, which were classified as Mottled-Dun pea by both models. So, while the violet colour factor had a significant role in distinguishing disease from speckling, it was not sufficient to discriminate the few samples that fell close to the classification border between their true class and the Mottled-Dun class. In a commercial setting this misclassification rate is acceptable since samples would first be screened for defects and only the non-defective grain would then be classified further into market grades. A third LDA model was developed to classify grain as defective or non-defective.

### Defect prediction Model

Differences in results for classifying defective samples through Model 1 and Model 2 indicated that a LDA model based on the same inputs would be able to also predict which field pea samples were defective among various market grade types. This proved true, all defective samples (both calibration and validation) were correctly classified (Table 4). All non-defective seed calibration samples were correctly classified and all but three samples of the non-defective seed validation set were correctly classified. While this model is not extended in the present study, the results indicate a modelling potential, to classify types of defects within grain samples, for example disease damage, insect damage and pre-harvest weather damage.

## Conclusion

Digital image analysis combined with linear discriminant analysis provides an effective tool for classifying pea market grades. In this study market grades of non-defective and defective seed samples were classified at 100% and up to 87% correctly, respectively. The choice of input variables influenced the robustness of the models to predict the market grades of defective as well as non-defective seeds. The input variables were based on industry-standard marketing traits, related to visual grain qualities, removing subjectivity through the application of digital image analysis.

## Supporting Information

S1 Fig Field pea market grades. a) White Pea, b) Blue Pea, c) Mottled Dun Pea, d) Kaspa Dun Pea, e) Green Dun Pea, f) Yellow Forage Pea, g) Marrowfat Pea, h) Kaspa-Type Pea.
(TIF)

S1 Table Descriptions of field pea market grades (DOCX)
