# Supplementary figures and images for: Discriminant Analysis of Defective and Non-Defective Field Pea (Pisum sativum L.) into Broad Market Grades Based on Digital Image Features
Source: PLoS One. 2016 May 13;11(5):e0155523. doi: 10.1371/journal.pone.0155523 (PMC4866801; doi:10.1371/journal.pone.0155523)

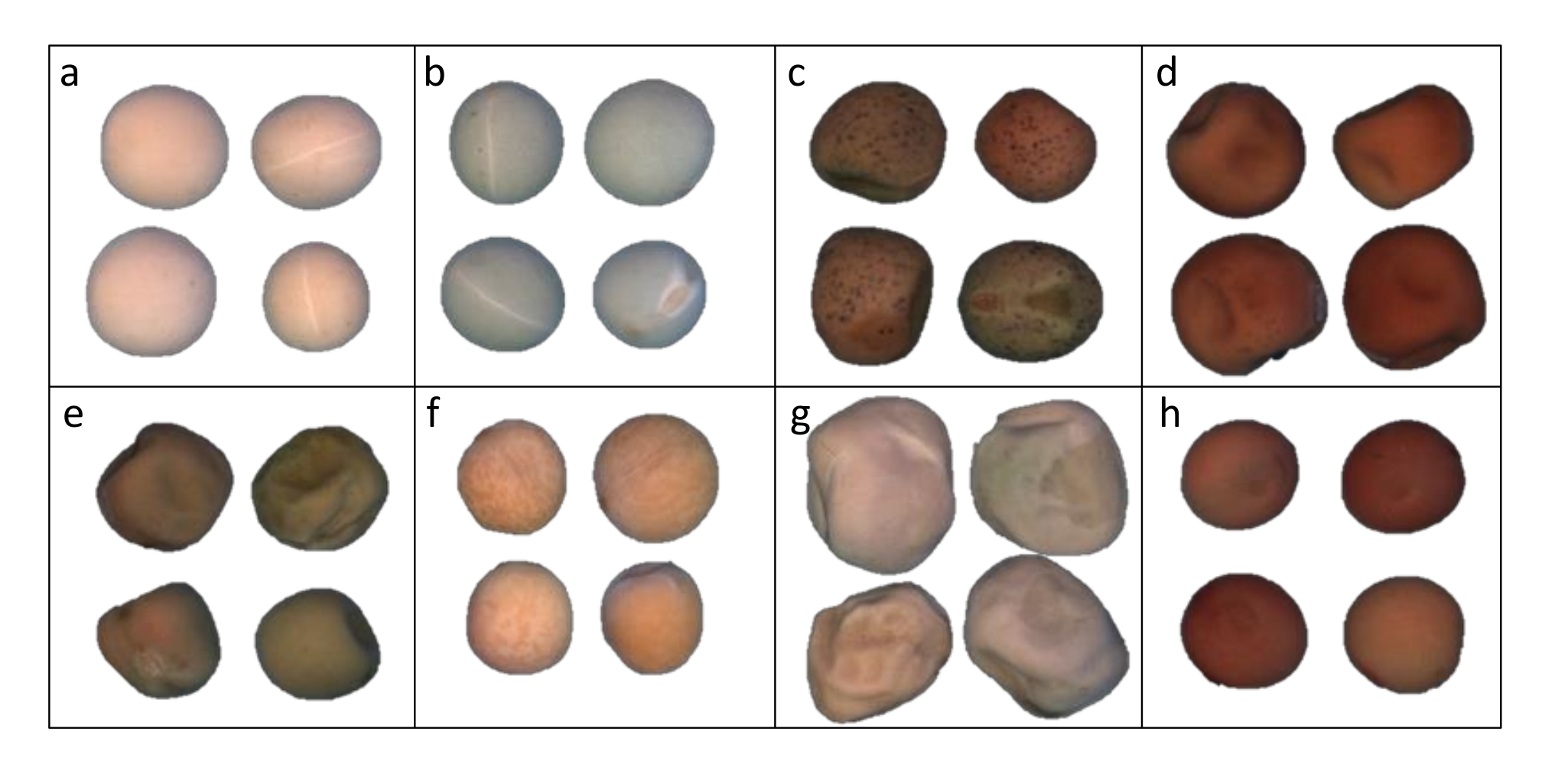

Supplement: S1 Fig — a) White Pea, b) Blue Pea, c) Mottled Dun Pea, d) Kaspa Dun Pea, e) Green Dun Pea, f) Yellow Forage Pea, g) Marrowfat Pea, h) Kaspa-Type Pea. (TIF) [file pone.0155523.s001.tif]
