# Supplementary material for: Discriminant Analysis of Defective and Non-Defective Field Pea (Pisum sativum L.) into Broad Market Grades Based on Digital Image Features
Source: PLoS One. 2016 May 13;11(5):e0155523. doi: 10.1371/journal.pone.0155523 (PMC4866801; doi:10.1371/journal.pone.0155523)
Supplement: S1 Table — (DOCX) [file pone.0155523.s002.docx]

| **White Pea** | **Blue Pea** | **Mottled Dun Pea** | **Kaspa Dun Pea** |
| --- | --- | --- | --- |
| Yellow cotyledon with white, opaque seed coat. Smooth spherical seed. | Green cotyledon with white-opaque seed coat. Smooth spherical seed. | Yellow cotyledon with a green/tan and speckled seed coat. Dimpled seed. | Yellow cotyledon with a tan coloured seed coat. Dimpled seed. |
| **Green Dun Pea** | **Yellow Forage Pea** | **Marrowfat Pea** | **Kaspa-Type Pea** |
| Yellow cotyledon with a tan and green coloured seed coat. Heavily dimpled seed. | Yellow cotyledon with opaque white seed coat. These look similar to white peas but are generally smaller in size and deeper yellow in colour. | Yellow cotyledon with white, opaque seed coat. Large, heavily dimpled seed | Yellow cotyledon with a tan coloured seed coat. Round seed with smooth seed coat (minimal dimples) |
